# Incidence of peripancreatic fluid collections in patients presenting with acute pancreatitis

**DOI:** 10.15537/smj.2022.43.12.20220508

**Published:** 2022-12

**Authors:** Nouran W. Molla, Abdullah H. Alsergani, Abduljabbar A. Alyamani, Majed A. Aljohani, Abdulaziz A. Aljohani, Fahad A. Alfaiz, Mohammed O. Alomar, Mohammed S. BinMayouf

**Affiliations:** *From the Department of Radiology (Molla), King Khalid University Hospital, King Saud University; and from the College of Medicine (Alsergani, Alyamani, Aljohani M, Aljohani A, Alfaiz, Alomar, BinMayouf), King Saud University, Riyadh, Kingdom of Saudi Arabia.*

**Keywords:** acute pancreatitis, peripancreatic fluid collections, incidence, CT scan, retrospective cohort

## Abstract

**Objectives:**

To calculate the incidence of acute peripancreatic fluid collection (APFC) in patients with acute pancreatitis. The secondary objective is to determine the underlying etiologies of acute pancreatitis in the Saudi population.

**Methods:**

A retrospective cohort study was carried out at King Khalid University Hospital, King Saud University, Riyadh, Saudi Arabia. The study analyzed data from patients who were diagnosed with acute pancreatitis between January 2008 and January 202. A total of 327 were included in the study after applying the inclusion and exclusion criteria. Their medical records were subsequently reviewed for the presence or absence of APFC on follow-up imaging studies, evidence of biliary stones, prior endoscopic retrograde cholangiopancreatography (ERCP), a history of alcohol use, and demographic variables.

**Results:**

Of the 327 patients with acute pancreatitis, 158 (48.3%) developed APFC, while 169 (51.7%) did not. The majority of patients had an idiopathic etiology of acute pancreatitis (n=251; 76.8%); followed by a biliary etiology (n=51; 15.6%); post-ERCP complications (n=14; 4.3%), and other causes (n=11; 3.3%).

**Conclusion:**

The incidence of APFC in patients presenting with acute pancreatitis between January 2008 and January 2021 was 48.3%. The most common etiology of acute pancreatitis in this tertiary care hospital was idiopathic, followed by biliary etiologies and post-ERCP complications. More studies targeting the local complications of pancreatitis are needed to reach more definitive findings.

**A**cute pancreatitis (AP) is characterized by the autodigestion of the pancreas by pancreatic enzymes, which are activated following the obstruction of the pancreatic ducts.^1^ Acute pancreatitis is associated with a high level of morbidity and mortality, with mortality rates reaching up to 10% of patients.^2^ The diagnosis of AP requires 2 of the following 3 features: abdominal pain consistent with AP, serum lipase or amylase activity (at least 3 times greater than the upper limit of normal), and characteristic findings of AP through contrast-enhanced computed tomography (CT), magnetic resonance imaging (MRI), or transabdominal ultrasonography (US).^3^ Alcohol consumption and biliary stone obstruction are the most common causes of AP internationally. However, there are a variety of less common structural, metabolic, and iatrogenic causes.^4,5^

The roles of early computed tomography (CT) scans in patients with AP include supporting the clinical diagnosis in equivocal cases, providing a prognostic indicator, and assessing complications. Therefore, they are usually conducted soon after admission.^6^ Local complications of AP include acute peripancreatic fluid collection (APFC), pseudocyst formation, acute necrotic collection, and walled-off necrosis. Acute peripancreatic fluid collection is a fluid collection that usually develops in the first 4 weeks of AP. Computed tomography scans show fluid collection around the pancreas in the absence of a fibrotic outer wall. Al though CT scans are the most common modality used for the detection of APFC’s, MRI’s and endoscopic US has been shown to be more accurate.^7,8^

Although APFC is mostly treated conservatively and resolves spontaneously, intervention is indicated when infection occurs. These interventions include percutaneous, endoscopic, and surgical drainage.^9^ A study by Kim et al^11^ found that among patients with alcoholic pancreatitis, those who developed APFC tend to have to have a severe course of AP with higher morbidity and mortality rates.^10^ Additionally, Kim et al^11^ found that patients with APFC had longer hospital stay compared to those with no fluid collection.

A study by Al-Lehibi et al^12^ found that admissions due to AP in a Saudi hospital were 35.7 admissions/year and biliary was the most common etiology. Earlier studies in Saudi Arabia at other tertiary care centers demonstrated an incidence of 5.2 admissions/year and 18.2 per year with similar etiological conclusions.^13,14^ Though inconclusive, these findings could indicate that the incidence of AP maybe trending upwards. As such more studies on AP are needed.

This study aims to identify the incidence of APFC in patients presenting with AP. Its hypothesis is that the incidence of APFC is going to be in most AP cases and that biliary pancreatitis is the most common etiology of AP

## Methods

This retrospective cohort study was carried out at King Khalid University Hospital, Riyadh, Saudi Arabia. The required data was gathered using an Electronic System for Integrated Health Information, Picture Archiving and Communication System, and a Radiology Information System (RIS). The inclusion criteria of this study are i) age above 18 years old, ii) patients diagnosed with AP, and iii) the availability of follow-up imaging within 4 weeks of admission. The exclusion criterion was that neither CTs nor MRIs were not carried out within 4 weeks of admission.

**Figure 1:**
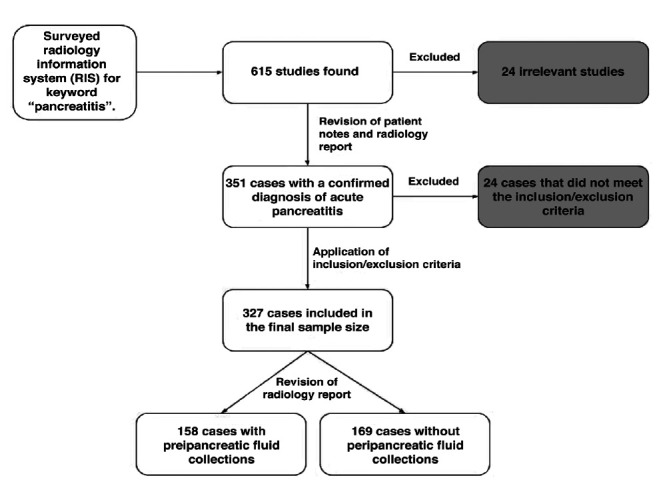
- Flowchart demonstrating sample selection process.

**Figure 2:**
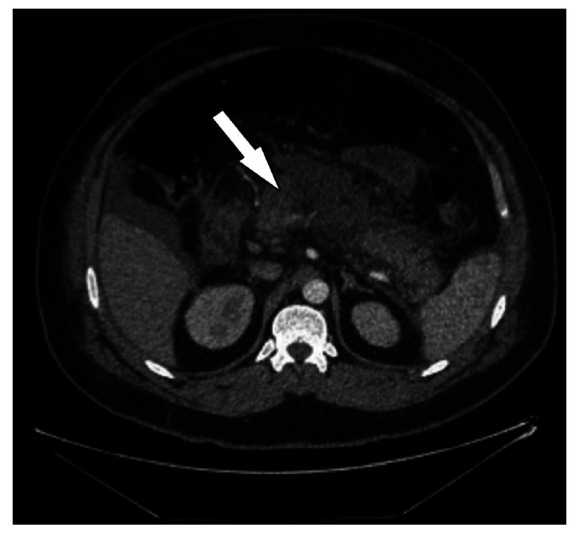
- Computed tomography scan of a patient with peripancreatic fluid collection (arrow).

**Figure 3:**
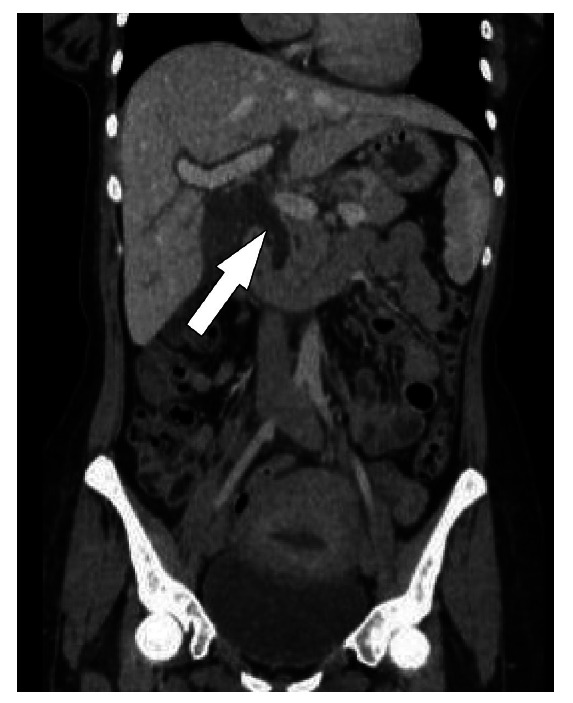
- Computed tomography scan of a patient with common bile duct dilation (arrow).

**Figure 4:**
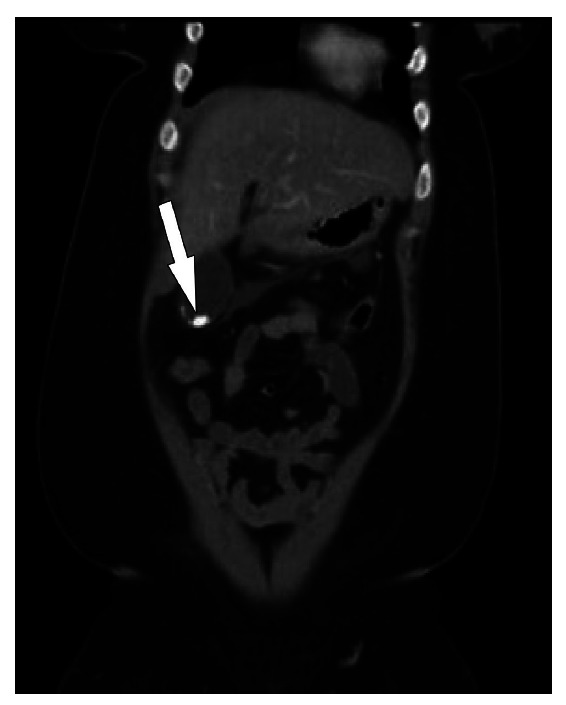
- Computed tomography scan of a patient with gallstones (arrow).

After obtaining ethical approval from the hospital’s Institutional Review Board, the keyword pancreatitis was used to search the RIS between January 2008 and January 2021, and 615 studies were collected. Patients without a confirmed AP diagnosis were excluded, resulting in 351 patients. Relevant inclusion and exclusion criteria were applied, which resulted in a sample of 327 patients. The process of sample selection is shown in Figure 1. Demographic data from the time of presentation included age, gender, and nationality is shown on Table 1. Details regarding AP included the presence or absence of a biliary stone, suspected etiology, and subsequent diagnostic and follow-up imaging studies for the presence or absence of APFC is shown in Table 2. An example of a patient with APFC is provided in Figure 2. Cases classified as biliary pancreatitis included patients with choledocholithiasis and patients with a dilated common bile duct (CBD), as indicated in Figure 3, and patients with gallstones in the gallbladder, as shown in Figure 4. Cases of pancreatitis caused by alcohol or endoscopic retrograde cholangiopancreatography (ERCP) were classified based on the patient’s medical history. Cases with an idiopathic etiology showed no evidence of these etiologies.

**Table 1:** - Demographic data of patients presenting with acute pancreatitis.

Characteristics	n	%
***Gender***		
Female	142	43.4
Male	185	56.4
Age (years), median (quartiles 1 & 3)	45	(34, 59)
***Age group***		
<45	153	46.8
45-65	114	34.8
>65	60	18.4
***Nationality***		
Saudi	303	92.7
Non-Saudi	24	7.3

**Table 2:** - Descriptive statistics of radiological data of patients presenting with acute pancreatitis.

Statustics	n	%
***Etiology of pancreatitis***		
Idiopathic	251	76.8
Biliary etiology	51	15.6
ERCP	14	4.3
Tumor	5	1.5
Other	6	1.8
***Imagine modalities***		
CT	234	71.6
MRI	93	28.4
***Peripancreatic fluid***		
No	169	51.7
Yes	158	48.3

ERCP: endoscopic retrograde cholangiopancreatography, CT: computed tomography, MRI: magnetic resonance imaging

### Statistical analysis

Our data was analyzed using the Statistical Package for the Social Sciences, version 24.0 (IBMCorp, Armonk, NY, USA). The histogram and the statistical Kolmogorov–Smirnov test were used to assess the normality assumption of continuous variables.

## Results

The demographic data of the patient population is presented in Table 1. The median age of the patients was found to be 45 years, 56.4% of whom were males. A total of 46.8% of patients were below 45 years old, while 34.8% and 18.4% were between 45-65 years old and above 65 years old. The sample of analyzed patients mainly consisted of Saudis (92.7%). Table 2 shows descriptive statistics on AP. CT scans comprised 71.6% of the modalities used, while MRIs comprised 28.4%. Of the 327 patients in the sample, 158 (48.3%) developed APFC, while 169 (51.7%) did not. The majority of AP cases (n=251; 76.8%) had idiopathic etiologies, followed by biliary etiologies (n=51; 15.6%), and (n=14; 4.3%) were post-ERCP complications.

## Discussion

The objectives of this study were to determine the incidence of APFC in patients presenting with AP and to determine the underlying etiologies of AP in the Saudi population. Patients under 45 years old comprised 46.8% of the total sample. A total of 34.8% of patients were 45-65 years old, and 18.4% were over 65 years old.

A total of 48.3% of patients developed APFC. This finding is consistent with international literature, in which multiple studies have shown an incidence of 30%–60%. Although this study’s incidence rate is at the higher level of this range, studies among Korean, German, and American populations had lower incidence rates of 42%, 37%, and 34%, respectively.^8,15,16^ However, the calculated incidence is not aligned with previous local studies, in which the incidence was 9% and 24%.^11,17^ This study’s higher incidence of APFC is likely because it only included patients with a severe enough disease course to undergo follow-up imaging therefor excluding patients with milder disease courses who did not have follow up imaging. Due to the varied incidence rates of peripancreatic fluid collection, many studies have been carried out to determine its predictors. Diculescu et al^18^ investigated many of these supposed predictors. Among them, only the presence of ascites was significantly associated with fluid collection. A study by Maringhini et al^5^ indicated that fluid collection occurred less frequently in patients with biliary pancreatitis than in those with other etiologies of pancreatitis. They also found that biliary pancreatitis was associated with a higher likelihood of a spontaneous resolution of fluid collection.^5^ This finding is similar to findings found on a study by Cui et al,^8^ which showed that alcoholic etiologies were more significantly associated with fluid collection than biliary etiologies. Older age and higher C-reactive protein levels 48 hours after admission were also found to be significant risk factors.

The study cohort’s most common etiology of AP was idiopathic, followed by biliary etiologies. These findings contradict most of the literature, in which biliary and alcoholic pancreatitis are the most common etiologies. The reason for this contradiction is that our main inclusion criteria was undergoing follow-up imaging within four weeks of presentation even if they have had ultrasounds. Many patients only received ultrasounds at presentation to determine the type of etiology without undergoing follow-up imaging, possibly limiting the inclusion of a significant number of AP cases with underlying biliary stone etiology. Therefore, this study was vulnerable to selection bias, as CT scans only detect a radio-opaque subtype of biliary stones and miss radiolucent stone subtypes. Interestingly, no cases of alcoholic pancreatitis were found among the collected data. A possible explanation for this is that alcohol is prohibited in Saudi Arabia, which, as other Saudi studies have found,reduces occurrences of alcoholic pancreatitis.^12-14,19^ Another is that patients did not disclose alcohol usage due to the sensitivity of this topic in Saud Arabia.

This study is significant for providing a baseline for how often clinicians can expect to find peripancreatic fluid collection, one of the prognostic indicators of the severity of pancreatitis, through follow-up imaging. That knowledge, in turn, may help predict the level of care a patient might require because AP cases with peripancreatic fluid collection have been associated with a more severe disease course than those without it.^8^

### Study limitations

Despite this study’s potential significance, it has limitations, such as a selection bias caused by limiting the inclusion of patients to those who had follow-up scans. This limitation led to more AP cases with idiopathic causes rather than biliary etiologies. The lack of ultrasound studies also contributed to this limitation. However, this was addressed by listing patients with a high likelihood of biliary etiology under biliary etiology. These patients included those with CBD dilation and gallstones who did not necessarily have choledocholithiasis. Another limitation was the poor documentation of alcohol use among patients, resulting in no cases being listed as alcoholic pancreatitis. Therefore, this study’s generalizability may have been limited.

In conclusion, APFC is one of many local complications contributing to the disease’s burden. This study found that peripancreatic fluid collection occurred in 48.7% of patients who presented with AP. It also found that the most common etiology of AP is idiopathic, followed by biliary etiology and ERCP-complicated pancreatitis. Studies investigating the risk factors for the development of APFC are necessary to explain the discrepancy in incidence rates found across multiple studies. More studies investigating peripancreatic fluid collections are needed to reach more solid conclusions. Accordingly, a follow-up study investigating the different risk factors associated with APFC will be performed on the same patient population and at the same hospital.
